# LARGE-SCALE ANALYSIS OF HUMAN GUT MICROBIOTA PATTERNS ACROSS GEOGRAPHY AND PEDIATRIC TO GERIATRIC AGES

**DOI:** 10.1093/geroni/igad104.2992

**Published:** 2023-12-21

**Authors:** Sherri Huang, Diptaraj Chaudhari, Pushti Kanani, Wesley Burrow, Yi Lin, Robert Mankowski, Shalini Jain, Hariom Yadav

**Affiliations:** University of South Florida, Tampa, Florida, United States; University of South Florida, Tampa, Florida, United States; University of South Florida, Tampa, Florida, United States; University of South Florida, Tampa, Florida, United States; University of Florida, Gainesville, Florida, United States; University of Florida, Gainseville, Florida, United States; University of South Florida, Tampa, Florida, United States; University of South Florida, Tampa, Florida, United States

## Abstract

Limited studies to-date have evaluated differences in gut microbiota across a global and temporal scale. Herein, we aim to examine the gut microbiota patterns across geography as defined by continent and countries and how gut microbiota vary across age groups within each geographic region. To approach these questions, we designed a large-scale analysis of gut microbiota across North America, Europe, Africa, Asia and Oceania. Literature across countries within each region was screened to include publications employing human stool samples and 16s rRNA sequencing of gut microbiota;116 publications resulted from this first screen. In the second screen, publications without metadata files or whose metadata files did not clarify sample type, collection timepoint or location were excluded. Raw 16s rRNA sequencing data for included publications were collected, and metadata for corresponding fastq files were screened to include only those of healthy, non-intervention groups. This final screen resulted a total of 25,665 metadata. Corresponding 16s rRNA gut microbiota sequences will be analyzed via the QIIME2 workflow in which we aim to compare and contrast gut microbiota and diversity across continents and countries and evaluate changes in gut microbiota from pediatric to geriatric age. This study will provide insight on biodiversity on a worldwide scale and inform on the changes in gut microbiota across time, allowing further examination of the role of the gut microbiome in the development of chronic disease in human aging. Altogether, our study represents one of the largest scale evaluation of gut microbiota patterns across geography and time.

